# Heterogeneous nuclear ribonucleoprotein hnRNPA2/B1 regulates the abundance of the copper-transporter ATP7A in an isoform-dependent manner

**DOI:** 10.3389/fmolb.2022.1067490

**Published:** 2022-12-05

**Authors:** Courtney J. McCann, Nesrin M. Hasan, Teresita Padilla-Benavides, Shubhrajit Roy, Svetlana Lutsenko

**Affiliations:** ^1^ Department of Physiology, Johns Hopkins University, Baltimore, MD, United States; ^2^ Department of Molecular Biology and Biochemistry, Wesleyan University, Middletown, CT, United States

**Keywords:** hnRNPA2/B1 regulates ATP7A abundance heterogeneous nuclear ribonucleoprotein (hnRNP), hnRNPA2/B1, ATP7A, copper, post-transcriptional regulation, SH-SY5Y cells, differentiation, Menkes disease

## Abstract

Copper (Cu) is an essential micronutrient with a critical role in mammalian growth and development. Imbalance of Cu causes severe diseases in humans; therefore, cellular Cu levels are tightly regulated. Major Cu-transport proteins and their cellular behavior have been characterized in detail, whereas their regulation at the mRNA level and associated factors are not well-understood. We show that the heterogeneous nuclear ribonucleoprotein hnRNPA2/B1 regulates Cu homeostasis by modulating the abundance of Cu(I)-transporter ATP7A. Downregulation of hnRNPA2/B1 in HeLa cells increases the *ATP7A* mRNA and protein levels and significantly decreases cellular Cu; this regulation involves the 3′ UTR of ATP7A transcript. Downregulation of B1 and B1b isoforms of hnRNPA2/B1 is sufficient to elevate *ATP7A*, whereas overexpression of either hnRNPA2 or hnRNPB1 isoforms decreases the *ATP7A* mRNA levels. Concurrent decrease in hnRNPA2/B1, increase in ATP7A, and a decrease in Cu levels was observed in neuroblastoma SH-SY5Y cells during retinoic acid-induced differentiation; this effect was reversed by overexpression of B1/B1b isoforms. We conclude that hnRNPA2/B1 is a new isoform-specific negative regulator of ATP7A abundance.

## Introduction

Copper (Cu) is an essential cofactor of enzymes involved in key physiological processes, including respiration, antioxidant defense, neurotransmitter production, and other important processes (Lutsenko, 2016; van den Berghe and Klomp, 2009; Uauy et al., 1998). Loss of Cu homeostasis causes diseases in both humans and animals (Uauy et al., 1998; Kaler, 2011). Menkes disease and Wilson disease are hallmark disorders of Cu misbalance, in which patients show systemic Cu deficiency or overload, respectively. Perturbations of Cu balance have been also found in patients with Alzheimer’s, Parkinson’s, and non-alcoholic fatty liver diseases (Waggoner et al., 1999; Kaler, 2011). To maintain Cu balance, cells possess a network of Cu-binding and transporting proteins that is regulated in response to metabolic cues and endocrine signals (Lutsenko, 2016). ATP7A is a key component of this Cu homeostatic network in most cells. ATP7A is a Cu(I)-transporting P_1B_-type ATPase that mediates dietary Cu uptake, Cu entry into the brain, and activation of cuproenzymes within the secretory pathway (Petris et al., 2002; Chun et al., 2017). ATP7A is located in the *trans*-Golgi network (TGN), and when intracellular Cu levels exceed cellular needs, it traffics *via* vesicles to the plasma membrane to export excess Cu from the cell (Lutsenko, 2016).

Regulation of ATP7A *via* trafficking has been studied in detail. The ATP7A kinase-mediated phosphorylation, the sequence motifs required for its endocytosis from the plasma membrane, and the return to the TGN have been described, along with proteins assisting ATP7A trafficking (van den Berghe and Klomp, 2010). By contrast, much less is known about the mechanisms by which ATP7A is regulated post-transcriptionally. Several studies described alternative mRNA splicing for *ATP7A* and a related *ATP7B* (Reddy and Harris, 1998; Harris et al., 2003; Milani et al., 2013; Lin et al., 2015). It was also shown that *ATP7A* mRNA processing may occur within the 3′ untranslated region (UTR) (Song et al., 2014; Vest et al., 2018; Xiao et al., 2018). The 3′ UTR of *ATP7A* is 3.8 kilobase pairs (kb) long—nearly as long as the 4.5 kb coding region—and more than one ATP7A transcripts differing in the length of their 3’ UTRs may exist (Vest et al., 2018). Despite the accumulating evidence for *ATP7A* mRNA being modulated, very few *trans*-acting factors, such as micro (mi)RNAs or RNA binding proteins, have been identified as candidates for *ATP7A* mRNA regulation (Song et al., 2014; Xiao et al., 2018).

hnRNPA2/B1 is a ubiquitous RNA-binding protein that is involved in alternative splicing, pre-mRNA packaging, trafficking, and nucleocytoplasmic shuttling of many transcripts (Shan et al., 2003; Friend et al., 2008; He et al., 2009; Han et al., 2010). hnRNPA2/B1 itself undergoes alternative splicing of exons 2 and 9, yielding four isoforms that are differentially expressed in a cell-specific manner. The roles of individual isoforms in various cellular processes are not well-understood. Interestingly, significant upregulation of the exons 2-containing B1 isoform(s) was observed in the liver of mice with Cu overload (Burkhead et al., 2011). This result suggested that hnRNPA2/B1 or one of the isoforms could be a Cu-responsive protein involved in Cu homeostasis. Here we tested this hypothesis.

We found that hnRNPA2/B1 regulates the abundance of *ATP7A* mRNA and protein *via* the mRNA 3′UTR in two different cell lines and that this effect is isoform specific. We also found that in neuroblastoma SH-SY5Y cells ATP7A abundance increased concurrently with a decrease in hnRNPA2/B1 during cells differentiation to regulate cellular copper levels.

## Materials and methods

### Cell culture

HeLa cells (ATCC^®^ CCL-2™) were cultured in complete medium consisting of Dulbecco’s modified Eagle’s medium (DMEM; Corning) supplemented with 10% heat-inactivated fetal bovine serum (FBS; Corning). Non-differentiated SH-SY5Y cells (ATCC^®^ CRL-2266™) were cultured in complete medium consisting of 1:1 Eagle’s Minimum Essential Medium (EMEM; Corning) and F12 Ham’s Medium (Corning) supplemented with 10% heat-inactivated FBS. All cells were maintained at 37°C in a humidified incubator with 5% CO_2_.

### Differentiation of SH-SY5Y cells

To differentiate, SH-SY5Y cells were plated at 20% confluency and treated as described previously (Kovalevich and Langford, 2013). Briefly, 24 h after plating the cells (day 0, d0), non-differentiated cells were collected and cells to be differentiated were treated with complete medium containing 10 µM retinoic acid (RA; Sigma). After 48 h of treatment (day 2, d2), the media was replaced with fresh media containing RA and incubated for a further 48 h. On day 4 (d4), the media was replaced with serum-free media containing 50 ng/ml brain-derived neurotrophic factor (BDNF; Shenandoah Biotech) and cells were incubated for 72 h before final collection on day 7 (d7).

### Small interfering (si)RNA-mediated knockdown

siRNA-mediated knockdowns were performed using wet-reverse transfection following DharmaFECT manufacturer’s protocols. Prior to transfection, all siRNA stocks were diluted to 5 µM in RNase-free water. Samples transfected without siRNA or non-targeting siRNA were used as controls. The sequences of siRNA (Dharmacon) used in this study are listed in Supplementary Table S1. Ten nanomoles of each siRNA (or an equivalent volume of medium) were incubated with DharmaFECT 1 transfection reagent (Dharmacon) in Opti-MEM reduced serum medium (Gibco) for 30 min at room temperature (RT). Cells grown to 40%–60% confluency were harvested and plated at a density of 2 × 10^5^ cells per well (6-well plates) or 6 × 10^5^ cells per well (6 cm plates), as indicated, in complete medium containing the siRNA-DharmaFECT mixture. After 48 h of incubation at 37°C, 1 ml of complete medium was added to each well, and the transfection was continued for 24 h more, for a total transfection time of 72 h.

For differentiated SH-SY5Y cells, cells were transfected with siRNA as described above. After 24 h of transfection, the media was aspirated and fresh complete medium containing 10 µM RA was added. Cells were incubated for 48 h more, for a total transfection time of 72 h, before collection.

### Generation of pSF-6xHis-GFP-TEV-hnRNPA2/B1 isoform plasmid constructs

His-tagged pET28a bacterial expression plasmids of each hnRNPA2/B1 isoform were kindly gifted by Dr. Max Konig. To insert the cDNA for each hnRNPA2/B1 isoform into the pSF-6xHis-GFP-TEV mammalian expression vector (Cat. Nos. OG4716 and OXGENE), the plasmids and the vector were digested with EcoRI and XhoI restriction enzymes for 3 h at 37°C and purified from a 0.8% agarose gel using a PureLink Quick Gel Extraction Kit (Invitrogen). hnRNPA2/B1 isoform inserts were ligated into the pSF-6xHis-GFP-TEV vector by incubating with T4 DNA ligase (New England Biolabs) for 2 h at RT. Ligated plasmids were transformed into 5α competent *E. coli* cells (New England Biolabs) following the manufacturer’s protocol. Plasmid DNA was isolated by miniprep using a QIAprep Spin Miniprep Kit (Qiagen). Sequences were confirmed using the primers listed in Supplementary Table S2.

### Transient transfection

Cells were grown to approximately 60% confluency in 6-well or 12-well plates, as indicated, and transfected with plasmid DNA using Lipofectamine LTX with PLUS reagent (Invitrogen) following the manufacturer’s protocols. For 6-well plates, a total of 2 µg plasmid DNA and 6 µl lipofectamine diluted in Opti-MEM were used per well; for 12-well plates, a total of 1 µg plasmid DNA and 3 µl lipofectamine were used. Fresh complete medium was added to the cells and the DNA-lipofectamine mixture added dropwise. The transfection was carried out for 20 h.

### Western blotting

Cells were lysed in 1X RIPA (10X: 0.5 M tris-HCl, pH 7.0; 1.5 M NaCl; 2.5% deoxycholic acid; 10% NP-40; 10 mM EDTA; Millipore) for 30 min on ice. Unless otherwise indicated, lysates were then centrifuged at 3,000 × g for 15 min to pellet cell debris. Protein concentrations were determined using BCA assay (Pierce). Twenty micrograms of protein were separated on 15% SDS gels; to achieve good resolution and separation of the individual hnRNPA2/B1 isoforms, gels were run until the 25 kDa ladder marker (PageRuler Plus Prestained Protein Ladder; Thermo Scientific) ran off the bottom of the gel. Protein was then transferred to PVDF (Millipore) membranes using 1X N-cyclohexyl-3-aminopropanesulfonic acid (CAPS) running buffer containing 10% methanol. Membranes were blocked for 1 h at RT in 5% milk in phosphate-buffered saline (PBS) and then incubated overnight (16 h) in the indicated primary antibodies (Supplementary Table S3) diluted in PBS, 0.2% Tween 20 (PBST) and 0.05% sodium azide. Membranes were washed three times in PBST and then incubated for 1 h at RT in the indicated secondary antibodies (Supplementary Table S3) diluted in 1% milk (PBST). Following two washes in PBST and one wash in PBS, membranes were treated with horseradish peroxidase (HRP) substrate for enhanced chemiluminescence (ECL; Pierce), and then imaged on an AlphaImager (ProteinSimple). The raw intensity of each band was quantified with ImageJ (NIH) and normalized to indicated loading controls. At least three biological replicates (*n* = 3) were performed for each condition. Relative protein abundances for the experimental samples were calculated by normalizing to loading controls and then to experimental controls, as indicated.

### Quantitative real-time polymerase chain reaction

Total RNA was isolated from cells using the RNeasy kit (Qiagen) following the manufacturer’s protocols. Quantitative real-time polymerase chain reaction (qRT-PCR) was performed using Power SYBR Green RNA-to-CT 1-Step Kit (Thermo Fisher) following the manufacturer’s instructions using 50 ng RNA per reaction on a QuantStudio 7 Flex Real-Time PCR machine (Applied Biosystems). At least three technical and biological replicates (*n* = 3) were performed for each condition: ex2 *n* = 15, ex9 *n* = 9, ex1_3 *n* = 9, ATP7A *n* = 21, ATP7B *n* = 6, Ctr1 *n* = 9, SOD1 *n* = 6. Primers used are listed in Supplementary Table S2. Fold-changes for the experimental samples were calculated by normalizing to hS18 loading control and then to sample controls, as indicated.

### Analysis of cellular copper content by atomic absorption spectrometry

HeLa cells following siRNA-mediated knockdown of *hnRNPA2/B1*, as well as proliferating and differentiating SH-SY5Y cells were grown in 6 cm dishes, washed three times in PBS, and split into two aliquots: one containing 10% of the cells and another containing 90% of the cells. The cells in the 10% aliquot were lysed in 1X RIPA buffer for 30 min on ice, centrifuged at 3,000 × *g* for 15 m, and the supernatant used to quantify total protein content by BCA assay. The cells in the 90% aliquot were digested in 200 µl of 70% nitric acid at 65°C for 2 h and, after cooling to RT, the sample volumes were adjusted to 500 µl using HPLC-grade water. Samples were stored at 4°C until analysis. Prior to measurement, samples were diluted 1:10 in HPLC-grade water and a standard Cu curve of 0, 10, and 100 parts per billion (ppb) Cu was constructed. Measurements were taken on a PinAAcle 900T atomic absorption spectrometer (Perkin Elmer). At least three biological replicates and three technical replicates were performed for each condition. Cu content values were normalized to total protein content, and results are reported as µg Cu/µg protein.

### Immunofluorescence analysis

Cells were grown to confluency on either untreated 18 mm glass coverslips (Electron Microscopy Sciences; for HeLa cells) or collagen-coated 18 mm glass coverslips (Neuvitro; for SH-SY5Y cells). After any treatments, cells were washed twice in PBS and then fixed in 4% paraformaldehyde (PFA; Electron Microscopy Sciences). Fixed cells were permeabilized in 0.1% Triton X-100 (Thermo Fisher) for 15 min at RT and then blocked in 5% bovine serum albumin (BSA; Gemini Bio-Products) for 40 min at RT. Coverslips were incubated in primary antibodies (Supplementary Table S3) diluted in 1% BSA for 1 h at RT and then washed twice in PBS + 0.2% tween (PBST) and once in PBS and incubated in secondary antibodies (Supplementary Table S3) diluted in 1% BSA for 1 h at RT. Coverslips were washed twice in PBST and once in PBS before being quickly rinsed in deionized water. Coverslips were mounted to glass slides with DAPI-Fluoromount (Electron Microscopy Sciences) and cured overnight. Images were taken with a Zeiss LSM800 microscope with a Plan-Apochromat 63x/1.4 NA oil lens and processed with ImageJ (NIH).

### Analysis of ATP7A trafficking

HeLa cells grown on glass coverslips were treated with media containing 0, 5, 10, or 20 µM CuCl_2_ for 4 h at 37°C. SH-SY5Y cells were grown on collagen-coated glass coverslips in 12-well plates and differentiated as described above. After treatment, cells were prepared for immunofluorescence analysis as described above. Anti-ATP7A and anti-TGN46 primary antibodies were used to examine ATP7A targeting to TGN and exit in response to Cu. Donkey anti-mouse AlexaFluor 488 and donkey anti-sheep AlexaFluor 568 secondary antibodies were used in immunostaining. The extent of ATP7A and TGN-46 colocalization (Pearson coefficient) was quantified using Coloc2 Plugin from ImageJ software.

### Dual luciferase assay

To assess the effect of *hnRNPA2/B1* knockdown, HeLa cells were transfected with non-targeting (NT), hnRNPA2/B1-total (total KD), or hnRNPA2/B1-ex2 (ex2 KD) siRNA in 6-well plates, as described above. Then, the cells were transfected with 1,200 ng of empty pcDNA plasmid, 600 ng *ATP7A* 3′UTR-luciferase plasmid (Cat. No. 128100810195; Applied Biological Materials, Inc.), and 200 ng Renilla-luciferase plasmid for a total of 2 µg of DNA. To assess the effect of hnRNPA2/B1 overexpression, HeLa cells were grown in 12-well plates and transfected with 600 ng of empty pcDNA plasmid, empty pSF-6xHis-GFP-TEV plasmid, or the individual pSF-6xHis-GFP-TEV-hnRNPA2/B1 isoform plasmids; 300 ng *ATP7A* 3′UTR-luciferase plasmid; and 100 ng Renilla-luciferase plasmid for a total of 1 µg of DNA transfected.

Following 20 h of transfection, cells were prepared for dual luciferase assay (Promega) following the manufacturer’s protocol. Briefly, cells were washed once with PBS and then incubated in either 500 µl (6-well) or 250 µl (12-well) 1X Passive Lysis Buffer (PLB) for 15 min at RT with shaking. Lysates were collected and 20 µl of each were dispensed into each well of a black, flat-bottom 96-well plate (Corning). Luminescence was measured on a FLUOStar Omega plate reader (BMG Labtech) using the following workflow: dispense 100 µl Luciferase Assay Buffer II reagent, wait 1 s, measure firefly luminescence in half-second intervals for 12 s, dispense 100 µl Stop & Glo reagent, wait 1 s, and measure Renilla luminescence in half-second intervals for 12 s. Firefly luminescence and Renilla luminescence reads over the 12 s measurements were summed up individually, and then total firefly luminescence was normalized to total Renilla luminescence. At least three technical and biological replicates were performed for each condition. Luminescence values of the experimental samples were normalized to the sample controls, as indicated.

### Statistical analysis

All values are reported as means ± standard deviation (SD) from at least three independent experiments. Statistical analyses were performed using Student’s *t*-test, one-way ANOVA, or two-way ANOVA, as indicated, using GraphPad Prism v8 (GraphPad Software, Inc.). *p < 0.05, **p < 0.01, ***p < 0.001 ****p < 0.0001.

## Results

### Downregulation of hnRNPA2/B1 decreases copper content in HeLa cells

**FIGURE 1:**
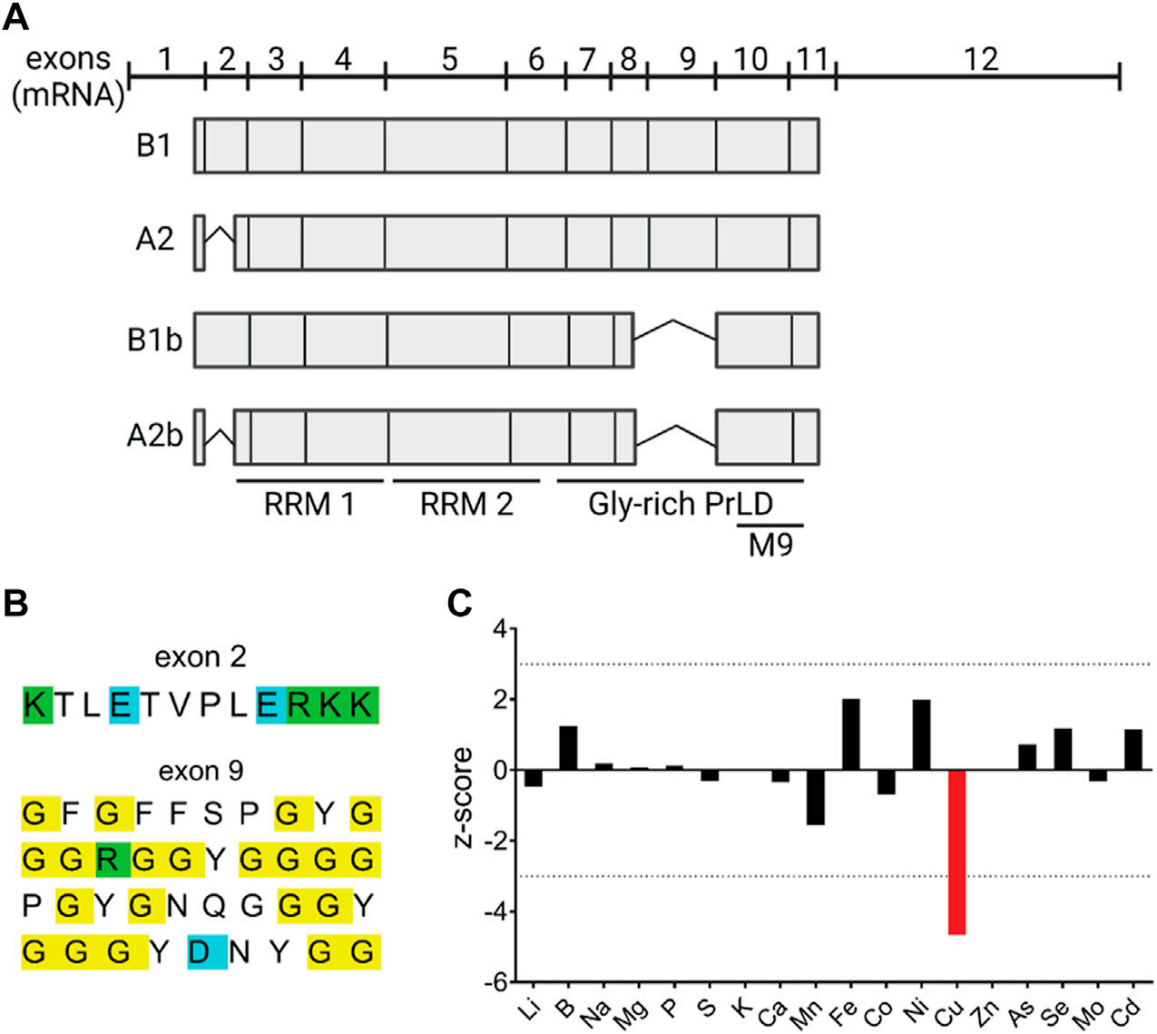
RNA-binding protein hnRNPA2/B1 modulates Cu levels in HeLa cells. **(A)** hnRNPA2/B1 has four distinct isoforms that arise from the alternative splicing of exons 2 and 9. Cartoon representation of the organization of the four hnRNPA2/B1 isoforms at the transcript-level. Exons 2 and 9 are alternatively spliced to generate the four isoforms: B1, A2, B1b, and A2b. Box length is indicative of the relative size of the exon within the transcript. RNA recognition motifs (RRMs), glycine-rich prion like domain (PrLD) **(B)** Amino acid sequences of exons 2 and 9. Positively charged amino acids are indicated in green, negatively charged amino acids in blue, and glycines in yellow. **(C)** Knockdown of *hnRNPA2/B1* in HeLa cells results in a specific and significant decrease in Cu levels. hnRNPA2/B1 expression was downregulated in HeLa cells *via* siRNA-mediated knockdown, and inductively coupled plasma mass spectrometry (ICP-MS) was used to measure the abundance of trace metals, including Cu, in these cells. Data are represented graphically by plotting the z-score, which indicates the number of standard deviations from the mean the signal value was based on the measurement of 3 replicates. A positive z-score indicates an increase and a negative z-score a decrease in the metal content (Malinouski et al., 2014).

The four isoforms of hnRNPA2/B1—the major A2 and B1 and the minor A2b and B1b—arise from alternative splicing (Figure 1A). The B1 isoform is the longest and includes all 11 protein-coding exons and 3′UTR; isoform B1b is missing exon 9 (ex9), isoform A2 is missing exon 2 (ex2), and A2b isoform lacks both ex2 and ex9. The isoforms are differentially expressed depending on cell types and/or cell cycle stage (Friend et al., 2008; Han et al., 2010). The ex2-containing isoforms of hnRNPA2/B1 (B1 and/or B1b) are enriched in hepatic nuclei in response to Cu accumulation suggesting that these isoforms may either directly sense Cu or otherwise regulate Cu balance (Burkhead et al., 2011). However, the amino acid sequences encoded by ex2 show no residues typically found in the Cu-binding sites, such as Cys, Met, and His (Figure 1B). Therefore, to examine whether hnRNPA2/B1 regulates Cu homeostasis, we first took advantage of the siRNA/metallomics dataset generated in our earlier study (Malinouski et al., 2014). In this work, the genome-wide siRNA screen identified proteins, down-regulation of which altered the content of specific metals in HeLa cells (Malinouski et al., 2014). Using this dataset, we found that downregulation of hnRNPA2/B1 decreased the Cu content of HeLa cells by approximately four standard deviations from the mean, while other metals were much less affected (Figure 1C).

**FIGURE 2:**
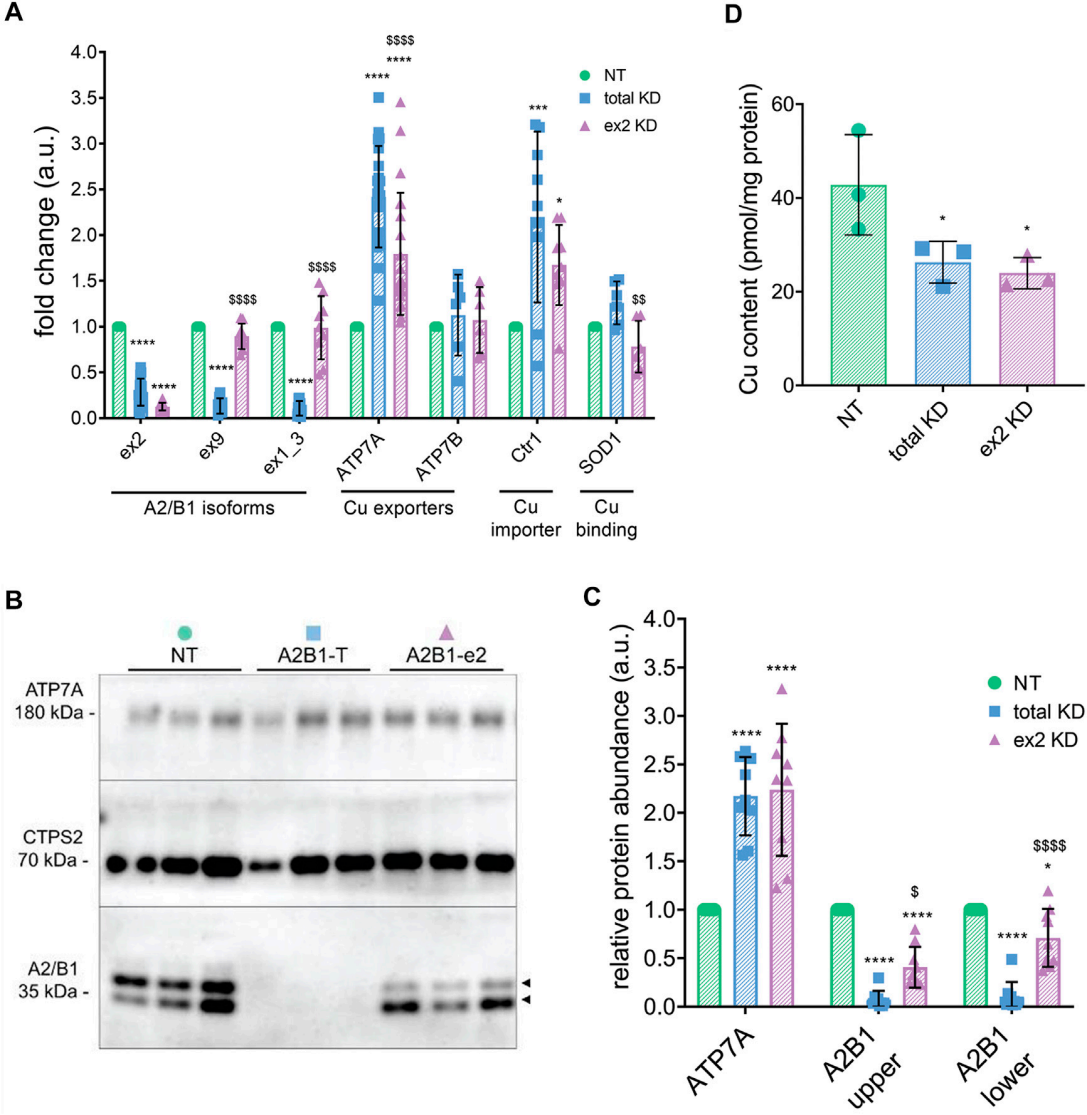
hnRNPA2/B1 downregulation in HeLa cells results in an increase in the abundance of the Cu(I) transporter ATP7A, and this effect is due in large part to the exon 2-containing B-isoforms. HeLa cells were transfected with non-targeting (NT) siRNA, siRNA against all 4 isoforms of hnRNPA2/B1 (total KD), or siRNA against the exon 2-containing B-isoforms of hnRNPA2/B1 (ex2 KD) for 72 h **(A)** qRT-PCR analysis of the mRNA abundance of the transcripts for Cu-handling proteins and hnRNPA2/B1 isoforms. Values normalized to hS18 and NT controls; exon 2 *n* = 15, exon 9 *n* = 9, exon 1_3 *n* = 9, ATP7A *n* = 21, ATP7B *n* = 6, Ctr1 *n* = 9, SOD1 *n* = 6. **(B)** Representative Western blot. Two distinct isoforms of hnRNPA2/B1 (upper B1 and lower A2) are detected, indicated by black arrowheads. **(C)** Densitometric analysis of protein abundance. Values normalized to CTPS2 and NT controls, *n* = 9. All values are reported as means ± SD. **(D)** Atomic absorption spectrometry analysis of cellular Cu content. Values normalized to protein content and NT control, *n* = 3. Significance was determined by two-way ANOVA; *p < 0.05, **p < 0.01, ***p < 0.001, ****p < 0.0001 compared to NT and ^$^ *p* < 0.05, ^$$^ *p* < 0.01, ^$$$^ *p* < 0.001 compared to total KD. Not-significant are not labelled.

To verify that hnRNPA2/B1 affects the cellular Cu content and to determine whether this effect was isoform-specific, hnRNPA2/B1 in HeLa cells was down-regulated using siRNA probes directed either against the sequence common for all four hnRNPA2/B1 isoforms (total KD) or only against the ex2-containing B isoforms (ex2 KD). (The targeted regions of hnRNPA2/B1 transcript are indicated in Supplementary Figure S1). A non-targeting (NT) siRNA was used as a control. The total KD significantly decreased the mRNA levels of all hnRNPA2/B1 isoforms (Figure 2A). Cells with the ex2 KD showed a 90% decrease of the ex2-containing transcripts and no change in the levels of ex9 and ex1-3 (exon 1 and 3 junction) containing transcripts, confirming specific downregulation of B-isoforms (Figure 2A). Western blot analysis of cell homogenates confirmed changes in protein abundance following hnRNPA2/B1 knock-downs. Control cells had two major hnRNPA2/B1 bands: One at approximately 40 kDa, consistent with the longer isoform B1 (predicted mass: 37.4 kDa), and a second band at approximately 35 kDa, consistent with the isoform A2 (predicted mass: 36 kDa) (Figure 2B). Both protein bands were significantly diminished in the total KD cells (Figures 2B,C). The ex2 KD cells had a marked decrease of only the upper band, confirming it was the B1-isoform, while the lower band (A2-isoform) was unchanged (Figures 2B,C). The minor isoforms B1b and A2b are not detected/or resolved in these experiments.

Analysis of the cellular Cu content by atomic absorption spectrometry found 38% less Cu in the total KD cells and 44% less Cu in ex2 KD cells, when compared to NT control (Figure 2D). The similar decrease in Cu levels by the total KD and ex2-specific KD suggested that the B-isoform(s) could be primarily responsible for this effect.

### hnRNPA2/B1-dependent upregulation of ATP7A explains the decrease in the cellular copper content

Cellular Cu content depends on Cu uptake, mediated by the high-affinity Cu transporter Ctr1 (SLC31A1), Cu efflux carried by the Cu-transporting ATPases ATP7A and/or ATP7B, and the abundant Cu-containing proteins, such as superoxide dismutase 1, SOD1. To identify which Cu-handling proteins were affected by hnRNPA2/B down-regulation, we analyzed the mRNA levels of ATP7A, ATP7B, Ctr1, and SOD1 (Figure 2A). Both total KD and ex2 KD cells showed no changes in ATP7B and SOD1 mRNA levels, whereas Ctr1 and ATP7A mRNA levels were upregulated in response to hnRNPA2/B1 downregulation. The increase in *ATP7A* mRNA led to a higher ATP7A protein abundance: a 1.9-fold increase in the total KD cells and a 2.1-fold increase in ex2 KD cells. Thus, B-isoform(s) of hnRNPA2/B1 modulate Cu levels in HeLa cells through changes in ATP7A and Ctr1 abundance. Given that the total effect was a decrease in cellular Cu content, which would not occur if Ctr1 import activity exceeds ATP7A efflux activity, we surmised that ATP7A plays a larger role in this phenotype and focused our attention on ATP7A.

### hnRNPA2/B1 downregulation in HeLa cells does not affect copper-dependent ATP7A trafficking

**FIGURE 3:**
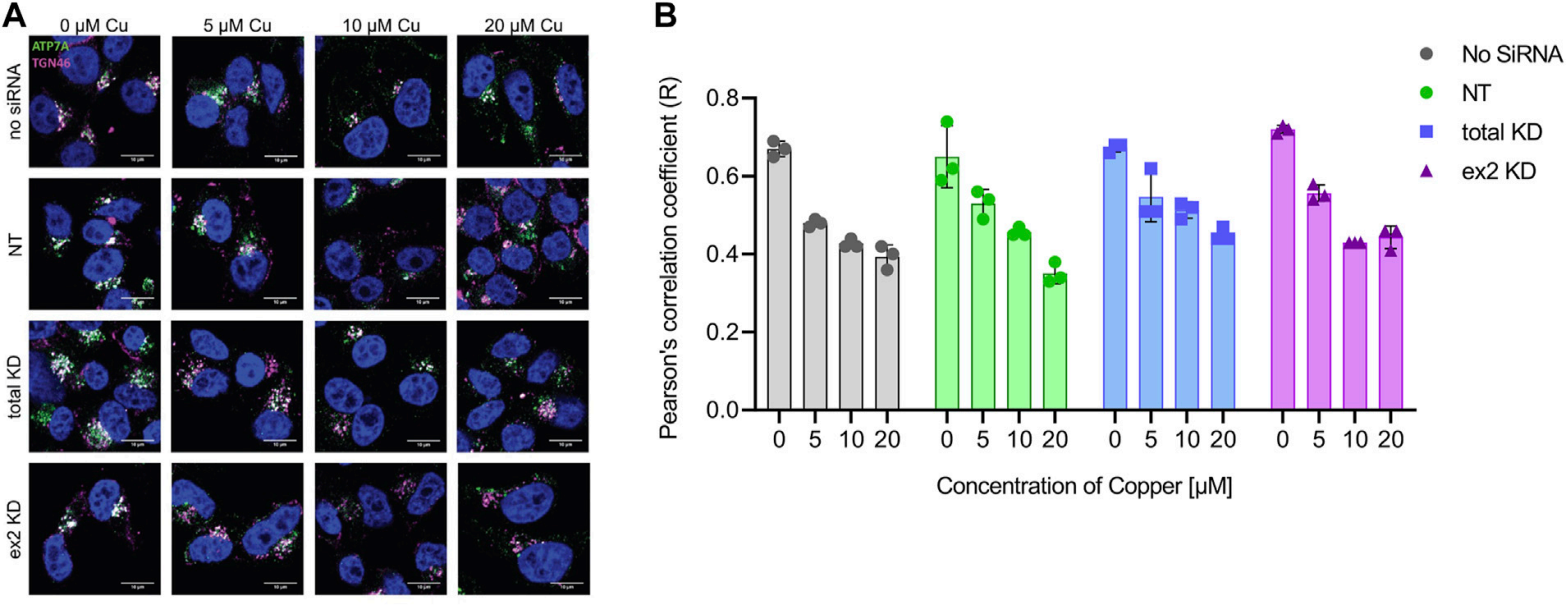
hnRNPA2/B1 downregulation in HeLa cells does not affect the trafficking of ATP7A in response to Cu. HeLa cells were transfected with no siRNA, non-targeting (NT) siRNA, siRNA against all 4 isoforms of hnRNPA2/B1 (total KD), or siRNA against the exon 2-containing B-isoforms of hnRNPA2/B1 (ex2 KD) for 72 h and then treated with the indicated concentrations of Cu for 4 h prior to staining **(A)** Immunofluorescence and confocal microscopy show that at basal levels, ATP7A is typically localized to the TGN and, upon Cu treatment, traffics to vesicles. Pattern of ATP7A staining relative to the TGN in response to Cu. ATP7A is stained in green, TGN marker TGN46 in magenta, and the nucleus (DAPI) in blue. Co-localization between ATP7A and TGN46 is indicated by white coloration. Size bar 10 µm **(B)** Pearson’s correlation coefficient (R) values for ATP7A and TGN46 colocalization show similar changes in response to increasing Cu concentration for the control (no siRNA, NT) and a total KD and ex-2 KD cells). R was calculated using Coloc2 plugin of ImageJ Software (*n* = 3 per condition).

ATP7A maintains cellular Cu levels by exporting excess Cu. This process depends on ATP7A trafficking from the TGN to the cell membrane, where Cu is excreted (Lutsenko, 2016). Since Ctr1 levels and, presumably, Cu uptake were increased in the hnRNPA2/B1 knockout cells, it was possible that lower Cu levels in these cells were caused by enhanced ATP7A trafficking that overcompensates for the Ctr1-dependent Cu uptake. To test this idea, the localization of ATP7A was examined in both total KD and ex2 KD cells (Figure 3). The loss of co-localization between ATP7A and a TGN marker was indicative of ATP7A trafficking in response to Cu (Figure 3A) and it was quantified using Pearson correlation coefficient (Figure 3B). In control cells (either no siRNA or NT siRNA), significant co-localization between ATP7A and the TGN marker, TGN46, was observed in basal Cu, as expected. Also, as expected, the colocalization of ATP7A with TGN46 decreased upon exposure to elevated Cu. This behavior of ATP7A was unaltered in either the total KD or ex2 KD cells, i.e., a similar loss of co-localization between ATP7A and TGN46 was observed in response to Cu elevation. Thus, hnRNPA2/B1 downregulation in HeLa cells does not impact ATP7A targeting to the TGN or its trafficking in response to elevated Cu. The observed decrease in cellular Cu is predominantly due to an increased ATP7A abundance.

### hnRNPA2/B1-mediated regulation of ATP7A mRNA involves the 3′ UTR

**FIGURE 4:**
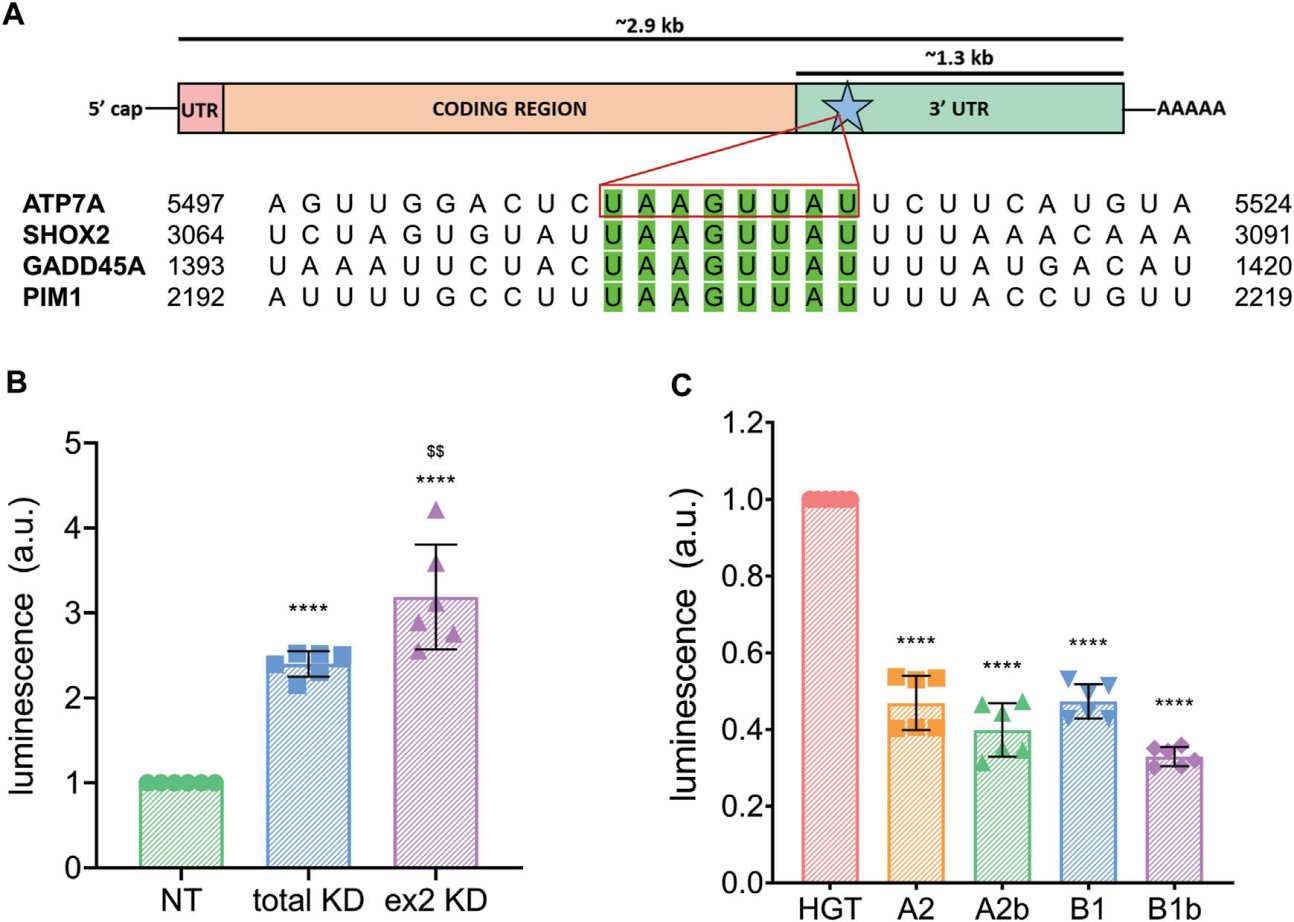
hnRNPA2/B1 regulates the expression of ATP7A through interaction with the transporter’s 3′ UTR. **(A)** Top, cartoon representation of the ATP7A transcript with the 5′ UTR indicated in pink, the coding region in orange, the 3′ UTR in green. The 8-nt motif known to direct hnRNPA2/B1-mediated decay is indicated with a blue star and green color. Box length is indicative of the relative size of these features. Bottom, sequence alignment of known hnRNPA2/B1 targets and ATP7A mRNA within the 8-nt motif. **(B)** Dual-luciferase assay showing that hnRNPA2/B1 downregulation results in an increase in ATP7A 3′ UTR expression. HeLa cells were transfected with non-targeting (NT) siRNA, siRNA against all 4 isoforms of hnRNPA2/B1 (total KD), or siRNA against the exon 2-containing B-isoforms (ex2 KD) for 48 h prior to transfection with ATP7A-3′ UTR-firefly luciferase, and Renilla-luciferase for 24 h (*n* = 6). **(C)** HeLa cells were co-transfected with the empty 6x His-GFP-TEV plasmid vehicle (HGT) or individual hnRNPA2/B1 isoforms at the same time as ATP7A-3′ UTR-luciferase and Renilla-luciferase plasmids, and dual-luciferase assay was carried out *n* = 6. All values are reported as means ± SD. Significance was determined by two-way ANOVA; *p < 0.05, **p < 0.01, ***p < 0.001, ****p < 0.0001 compared to NT, HGT, or neg, as indicated, and ^$$^ *p* < 0.01 compared to total KD.

hnRNPA2/B1 was shown to regulate many transcripts through interaction with an 8-nucleotide motif present in their 3′ UTRs (Geissler et al., 2016). The hnRNPA2/B1 binding at this site causes decay of the target mRNAs, whereas downregulation of hnRNPA2/B1 increases transcript levels. Analysis of the *ATP7A* mRNA sequence identified one copy of this motif (UAAGUUAU) near the beginning of its 3′-UTR (Figure 4A). The CTR1 transcript, which was also sensitive to downregulation of hnRNPA2/B1 (Figure 2A), does not have the exactly same motif, but does have a similar UAAGUAAAU sequence in its 3′-UTR.

To directly test whether the 3′-UTR of ATP7A mRNA is involved in the hnRNPA2/B1-mediated regulation of ATP7A, control HeLa cells, total KD and ex2 KD cells were transfected with a plasmid encoding firefly luciferase cDNA fused with the *ATP7A* 3′-UTR and a reference plasmid encoding Renilla luciferase. The abundance of the *ATP7A* 3′-UTR transcript was quantified through firefly luciferase luminescence normalized to Renilla luciferase signal. We predicted that luminescence would increase in cells with hnRNPA2/B1 knockdown if the *ATP7A* 3′-UTR is involved in hnRNPA2/B1-mediated regulation. Indeed, luciferase signal was 2.4 higher in the total the KD and 3.9 fold higher in the ex2 KD cells, compared to NT control (Figure 4B). Thus, the 3′-UTR of *ATP7A* mRNA plays a role in the hnRNPA2/B1-mediated regulation of ATP7A abundance, and the B-isoforms are involved in this process.

To test whether hnRNPA2/B1 overexpression would decrease *ATP7A* mRNA levels, individual hnRNPA2/B1 isoforms were cloned into a vector containing a GFP tag, and their expression was confirmed by the presence of GFP signal (Supplementary Figures S2A,B). A2b and B1 isoforms were targeted primarily to the nucleus, whereas A2 and B1b isoform were seen mostly in the cytosol (Supplementary Figure S2). Overexpression of B isoforms resulted in the decreased abundance of ATP7A 3′-UTR, as determined by the lower signal in dual-luciferase assay (Figure 4C). This result further supported the involvement of the B isoforms in the regulation ATP7A mRNA. Unexpectedly, overexpression of A2 isoforms also decreased the ATP7A 3′-UTR abundance. This suggested a more complex relationship between the hnRNPA2/B1 isoforms and ATP7A transcript levels, although non-specific effects of A2/A2b overexpression on ATP7A mRNA cannot be excluded due to high structural similarity between the isoforms.

### Opposite changes in ATP7A and hnRNPA2/B1 levels during differentiation of SH-SY5Y cells

**FIGURE 5:**
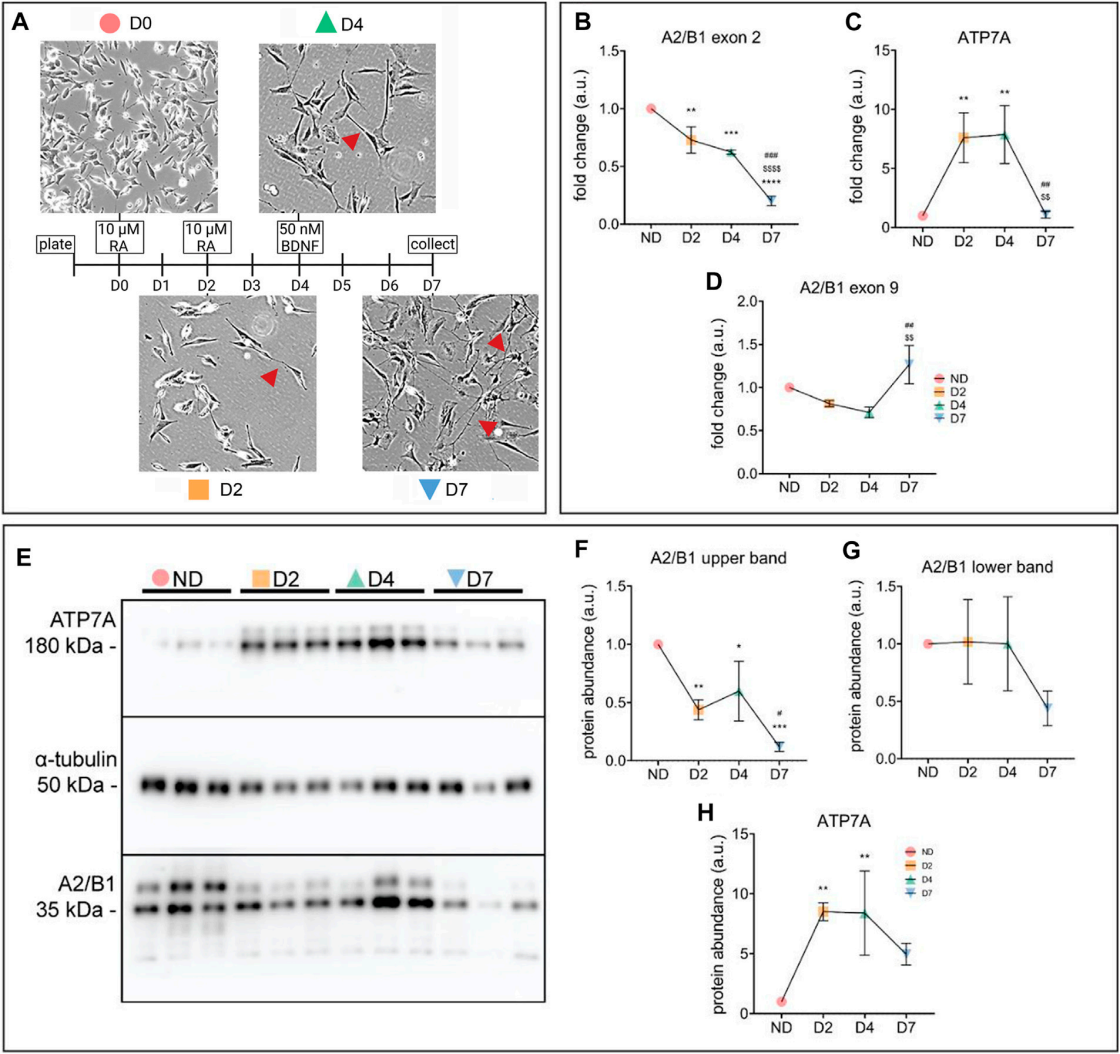
Concurrent increase of ATP7A and decrease of hnRNPA2/B1 occurs during neuronal differentiation in SH-SY5Y cells. **(A)** Schematic representation of SH-SY5Y differentiation using treatments with RA and BDNF. Cells were collected for analysis on days d0 (non-differentiated, ND), d2 (differentiated d2, D2), d4 (differentiated d4, D4), and d7 (differentiated d7, D7). Morphological changes in SH-SY5Y cells upon differentiation were observed *via* light microscopy. Neurites, an indication of neuronal maturation, are indicated with red arrowheads **(B–D)** qRT-PCR analysis of mRNA abundance of hnRNPA2/B1 isoforms **(B,C)** and ATP7A **(D)**. Values normalized to hS18 and ND controls, *n* = 3. **(E)** Representative Western blot showing two distinct isoforms of hnRNPA2/B1, indicated by black arrows **(F–H)** Densitometric analysis of protein abundance of hnRNPA2/B1 isoforms **(F,G)** and ATP7A **(H)**. Values normalized to α-tubulin and ND controls, *n* = 3. All values are reported as means ± SD. Significance was determined by two-way ANOVA; *p < 0.05, **p < 0.01, ***p < 0.001, ****p < 0.0001 compared to ND, ^$$$$^ *p* < 0.001 compared to D2, and ^###^ *p* < 0.001 compared to D4.

While the results thus far made it clear that hnRNPA2/B1 affects the ATP7A abundance in HeLa cells, it was less clear whether this regulatory relationship occurs in other cellular contexts or whether it plays a role in specific cellular processes. The expression of both *ATP7A* and *hnRNPA2/B1* is responsive to retinoic acid (RA), with *ATP7A* expression increasing in response to RA and *hnRNPA2/B1* expression decreasing in response to RA treatment (Zhou et al., 2001; Bohlken et al., 2009; Hatori et al., 2016). RA is known to induce neuronal differentiation (Hatori et al., 2016). Consequently, to determine whether hnRNPA2/B1-mediated regulation of *ATP7A* occurred during neuronal differentiation, SH-SY5Y neuroblastoma cells were differentiated using a two-step protocol using RA and then BDNF treatments (Figure 5). Cells were collected at four different time points during differentiation: before differentiation (ND), after 2-days of RA treatment (D2), after 4-days of RA treatment (D4), and after 4 days of RA treatment followed by 3-day BDNF treatment (D7). During this process, SH-SY5Y cells undergo morphological changes, with branched neurites starting to form at D2 and being fully differentiated at D7 (Figure 5A).

The mRNA levels of the ex2-containing *hnRNPA2/B1* isoforms decrease significantly over the course of SH-SY5Y differentiation (Figure 5B), while ATP7A expression first increased at D2, remained high at D4 and then decreased at D7 (Figure 5C). Unlike the ex2-containing B-isoforms, the abundance of exon 9-containing A-isoforms decreased slightly at D2 and D4, and was elevated at D7, potentially explaining the drop in ATP7A levels (Figure 5D). Overall, the protein abundance of hnRNPB1 and ATP7A was in agreement with the mRNA changes at D2, but not at the later stages (Figures 5E–G), suggesting that down-regulation of B1 isoforms impacts ATP7A early in differentiation, whereas other factors including A2 may play regulatory roles later.

**FIGURE 6:**
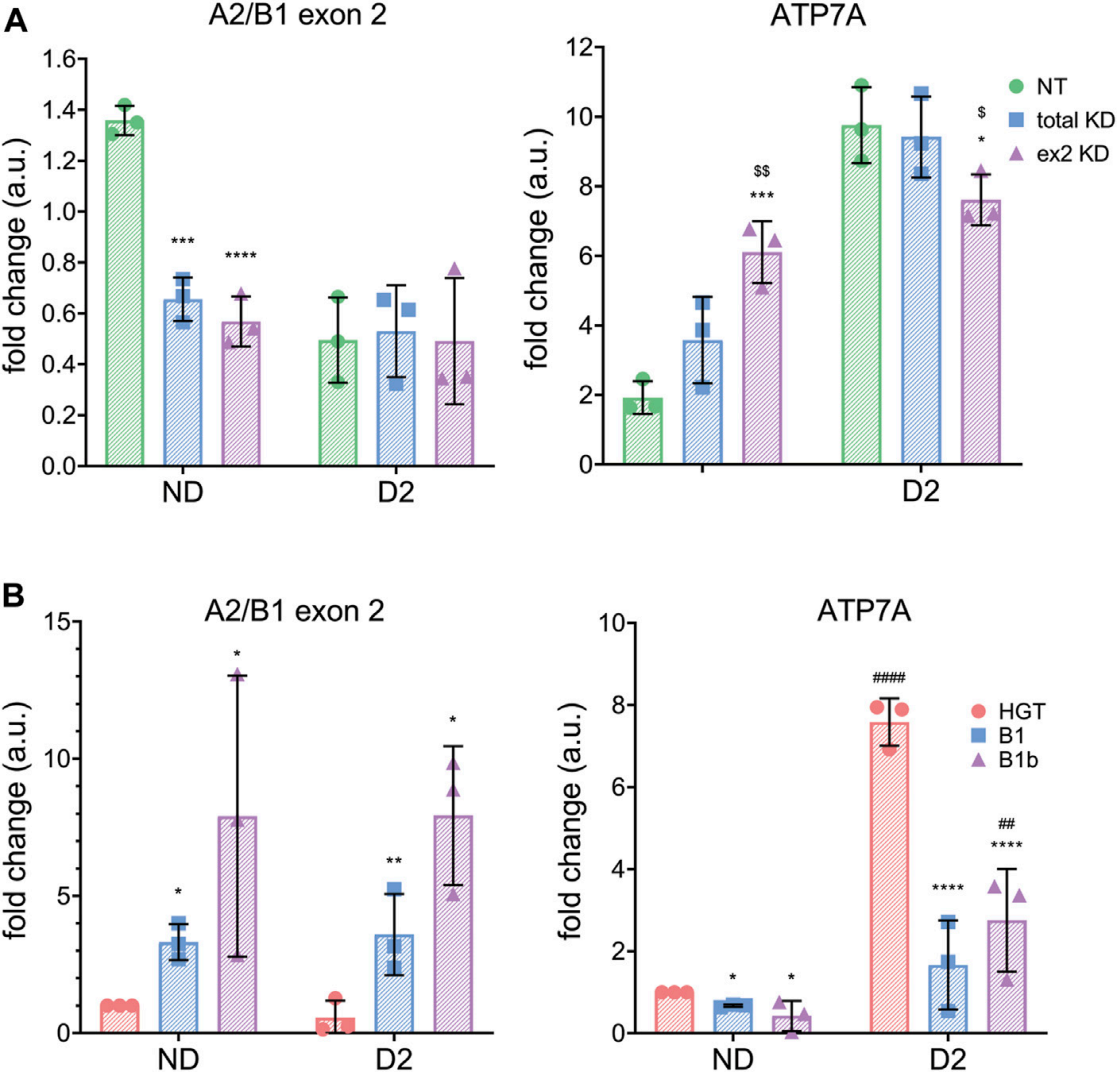
hnRNPA2/B1 regulates ATP7A expression in SH-SY5Y cells. SH-SY5Y cells were transfected with non-targeting (NT,green) siRNA, siRNA against all 4 isoforms of hnRNPA2/B1 (total KD, blue), or siRNA against the exon 2-containing B-isoforms of hnRNPA2/B1 (ex2 KD, purple). After 24 h of incubation, differentiation was induced by adding RA to the media. After 48 h, cells were collected for analysis. **(A)** qRT-PCR analysis of mRNA abundance of *hnRNPA2/B1* isoforms and *ATP7A* **.** Values normalized to hS18 and ND controls, *n* = 3. **(B)** *ATP7A* mRNA abundance decreases upon overexpression of exon 2-containing B-isoforms (green -empty plasmid, blue isoform B1 and purple-isoform B1b). qRT-PCR analysis of mRNA abundance of *hnRNPA2/B1* isoforms and *ATP7A* in non-differentiating and differentiating SH-SY5Y cells**.** Significance was determined by two-way ANOVA; *p < 0.05, **p < 0.01, ***p < 0.001, ****p < 0.0001 compared to NT and ^$^ *p* < 0.05, ^$$^ *p* < 0.01, ^$$$^ *p* < 0.001 compared to total KD.

To more directly test the link between the B-isoform abundance and ATP7A levels early in SH-SY5Y differentiation, *hnRNPA2/B1* was first downregulated in SH-SY5Y cells using total KD or ex2 KD siRNA and then differentiation was induced by treatment with 10 µM RA for 2 days. As previously observed in HeLa cells, downregulation of all or only the ex2-containing B1 isoforms led to an increase in the *ATP7A* transcript in non-differentiated (ND) cells (Figure 6A). In differentiating cells (D) cells, ATP7A mRNA was equally upregulated in control cells and in cells with downregulated hnRNPB1. This result suggested that the increase in ATP7A mRNA at D2 of differentiation is likely to be caused by hnRNPB1 downregulation. To further test this hypothesis, the B1 or B1b isoforms were overexpressed and differentiation was induced in a similar manner (Figure 6B). Overexpression of either the B1 or B1b isoforms decreased the levels of ATP7A mRNA at D2 compared to the mock-transfected control. Taken together, the data suggest that the change in hnRNPB1 expression modulate *ATP7A* levels during RA-induced neuronal differentiation.

**FIGURE 7:**
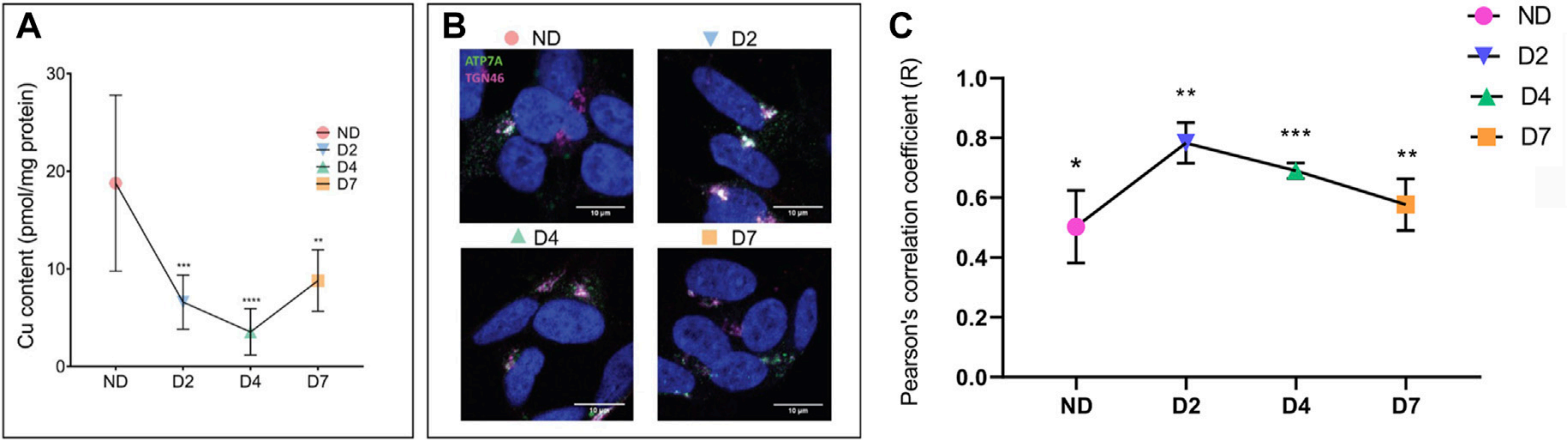
Cellular Cu decreases and ATP7A localization changes during neuronal differentiation. SH-SY5Y neuroblastoma cells were differentiated by treatments with RA and BDNF. Cells were collected for analysis on days d0 (non-differentiated, ND), d2 (differentiated d2, D2), d4 (differentiated d4, D4), and d7 (differentiated d7, D7). **(A)** Atomic absorption spectrometry (AAS) analysis of cellular Cu content. Values normalized to protein content and ND control, *n* = 6. **(B)** Pattern of ATP7A staining and **(C)** calculation of Pierson correlation coefficient (c) illustrates tighter TGN localization in response to Cu decrease. ATP7A is stained in green, TGN marker TGN46 in pink, and the nucleus (DAPI) in blue. Co-localization between ATP7A and TGN46 is indicated by white color. Pearson’s correlation coefficient was calculated using Coloc2 plugin of ImageJ software (*n* = 3). T-test was performed using Graphpad prism version 9.4.0. Data were represented as Mean ± SD; *p*-value *p < 0.05, **p < 0.01, ***p < 0.001.

Finally, we examined consequences of these changes on cellular copper homeostasis by measuring cellular Cu content in SH-SY5Y cells during differentiation (Figure 7A). AAS analyses of total cell extracts showed that Cu levels largely reflected changes in ATP7A, i.e. the Cu content decreased significantly at D2 and D4 and then increased as ATP7A levels diminished. Analysis of ATP7A trafficking at each of these points (Figures 7B,C) revealed that the localization of the transporter was consistent with changes in Cu levels. A more vesicular staining of ATP7A and a lower Pearson coefficient were seen when Cu was higher, whereas the co-localization with TGN46 was highest when Cu was lowest, at D2 (Figures 7B,C). Thus, hnRNPA2/B1-dependent changes of ATP7A abundance modulate available cellular Cu in SH-SY5Y during differentiation.

## Discussion

In this study we provide evidence that the RNA binding protein hnRNPA2/B1 is a new regulator of Cu homeostasis that modulates cellular Cu levels through regulation of ATP7A mRNA and protein levels. This process is both hnRNPA2/B1 isoform-specific and *ATP7A* 3′ UTR-dependent. The exon 2-containing B-isoforms of hnRNPA2/B1 mediate negative regulation of *ATP7A* abundance, whereas A2 isoform, when in excess, appears to have an opposite effect. The N-terminal 12 amino-acid residues of hnRNPB1 differentiate the B1 isoform from a highly similar hnRNPA2, and therefore this region is primarily responsible for the specific regulatory effects of hnRNPB1 on the ATP7A transcript. Although the AlphaFold2-generated structure of hnRNPB1 suggests that the exon 2-encoded protein region is unstructured (https://alphafold.ebi.ac.uk/entry/P22626), it is likely to be involved in specific protein interactions, which affects the isoforms localization in a cell: the hnRNPB1 isoform has a predominantly nuclear localization, whereas hnRNPA2 shows both nuclear and cytosolic localization (Supplementary Figure S2). The results also suggest that hnRNPB1 may retain the ATP7A transcript in the nucleus (limiting protein translation in the cytosol), whereas the hnRNPB1 downregulation would allow ATP7A mRNA to be transported out of the nucleus for translation and increased protein production.

The hnRNPA2/B1-mediated regulation of mRNA occurs in many forms: *via* alternative splicing, mRNA trafficking and localization-dependent translation, as well as maintenance of mRNA abundance *via* transcript stabilization/degradation (Munro et al., 1999; Kosturko et al., 2006; He et al., 2009; Moran-Jones et al., 2009; Han et al., 2010; Huelga et al., 2012; Geissler et al., 2016). Geissler et al. (2016) characterized an hnRNPA2/B1-mediated regulatory mechanism in which hnRNPA2/B1 binds to a specific 8-nt motif (UAA[G/C]UUAU) in the 3′ UTR of transcripts, leading to their destabilization *via* CCR4-NOT-directed deadenylation and subsequent decay. *ATP7A* mRNA has a long 3′ UTR, the functional significance of which is poorly understood, although recent studies have identified mechanisms by which changes in the length of the 3′ UTR may be involved in the regulation of *ATP7A* abundance (Song et al., 2014; Vest et al., 2018). Our work provides additional information about the role of this region. We found that *ATP7A* 3′ UTR has one copy of the UAAGUUAU hnRNPA2/B1-binding motif at the proximal end of the UTR, that the 3′ UTR of *ATP7A* is involved in hnRNPA2/B1-mediated regulation, and that, again, the B-isoforms play the main role in this process. Further studies are needed to fully delineate the ATP7A-3′ UTR determinants responsible for hnRNPB1 effects and determine whether the hnRNPB1 N-terminus directly or indirectly interacts with the UAAGUUAU sequence.

Previous studies have shown that hnRNPA2/B1 is involved in the maintenance of an undifferentiated phenotype of human embryonic stem cells and inhibits differentiation in several cell types (He et al., 2009; Choi et al., 2013). We demonstrate that early steps of differentiation of SHSY-5Y cells are associated with the decrease in hnRNPA2/B1 expression, which coincides with ATP7A upregulation. Reversal of *ATP7A* upregulation by overexpression of hnRNPB1/B1b strongly suggest that the levels of *ATP7A* mRNA and hnRNPB1 are linked. It is interesting that these changes during early neuronal cells differentiation are associated with transient lowering of intracellular copper. Previous studies found that elevated Cu increases cell proliferation and cell motility. It is tempting to speculate that a transient Cu decrease may be an integral component of cellular differentiation program that hinders proliferation. Future studies will establish the molecular consequences of transient Cu decrease in the cytosol and its role in cell differentiation.

Monitoring of *hnRNPA2/B1* mRNA shows that the abundance of ex2-containing transcripts decreases significantly over the course of differentiation, continuing to decrease even beyond the RA treatment to a minimum at D7. The ex9-containing isoforms, by contrast, decreased more modestly in response to RA treatment, and by D7 were even present at slightly higher levels compared to ND. This increase in ex9-containing isoforms could potentially be explained by auto-regulation of the *hnRNPA2/B1* mRNA by the resulting protein. Overexpression of the A2 isoform has been shown to reduce the abundance of A2 and B1 mRNA through binding the 3′ UTR and initiating an alternative splicing event that results in the nonsense-mediated decay of the transcripts (McGlincy et al., 2010). It is conceivable that the decreasing the amount of one hnRNPA2/B1 isoform could result in an upregulation of the other(s) due to the loss of this basal negative regulation of transcripts. While the potential interplay between the individual hnRNPA2/B1 isoforms will need further study, our data so far show that the inverse relationship between hnRNPA2/B1 and ATP7A expression occurs during neuronal differentiation and support the primary role B-isoforms in the regulation of ATP7A expression, and subsequently, cellular Cu levels. Taken together, our results provide a basis for future studies into the finer details of the post-transcriptional regulation of Cu homeostasis, especially in the context of neuronal differentiation.

## Data Availability

The original contributions presented in the study are included in the article/Supplementary Material, further inquiries can be directed to the corresponding author.
