# Supplementary material for: Heterogeneous nuclear ribonucleoprotein hnRNPA2/B1 regulates the abundance of the copper-transporter ATP7A in an isoform-dependent manner
Source: Front Mol Biosci. 2022 Dec 5;9:1067490. doi: 10.3389/fmolb.2022.1067490 (PMC9762481; doi:10.3389/fmolb.2022.1067490)
Supplement: Supplementary file 1 [file DataSheet1.PDF]

SUPPLEMENTARY MATERIALS

Heterogeneous Nuclear Ribonucleoprotein HnrnpA2/B1 Regulates the Abundance of the Copper-Transporter ATP7A in an Isoform-Dependent Manner

Courtney J. McCann[1,2], Nesrin Hasan[1], Teresita Padilla-Benavides[2], and Svetlana Lutsenko[1]
[1]Department of Physiology, Johns Hopkins University, Baltimore, MD, USA
[2]Department of Molecular Biology and Biochemistry, Wesleyan University, Middletown, CT, 06459, USA

SUPPLEMENTARY TABLES

Suppl. Table 1. Sequences of small interfering siRNA used in *hnRNPA2/B1* knockdown experiments.

siRNA	Catalog No.	Sequence
siGENOME non-targeting siRNA pool #1	D-001206-13	5’- UAGCGACUAAACACAUCAA -3’
		5’- UAGCGACUAAACACAUCAA -3’
		5’- AUGUAUUGGCCUGUAUUAG -3’
		5’- AUGAACGUGAAUUGCUCAA -3’
siGENOME hnRNP A2/B1 total siRNA pool	M-011690-01	5’- GGAGAGUAGUUGAGCCAAA -3’
		5’- GUUCAGAGUUCUAGGAGUG -3’
		5’- GAACAAUGGGGAAAGCUUA -3’
		5’- GCAAGACCUCAUUCAAUUG -3’
siGENOME hnRNP A2/B1-ex2 siRNA	Custom	5’- CUUUAGAAACUGUUCCUUUUU -3’

Suppl. Table 2. Sequences of primers used for plasmid sequences and qRT-PCR.

Primers	Sequence
**qRT-PCR primers**	
hnRNPA2/B1_ex1_3 F	5’- CGATGGAGAGAGAAAAGGAAC -3’
hnRNPA2/B1_ex1_3 R	5’- GCTTTCCCCATTGCTCATAG -3’
hnRNPA2/B1_ex2 F	5’- AACTTTAGAAACTGTTCCTTTGG -3’
hnRNPA2/B1_ex2 R	5’- CAGTCTGTAAGCTTTCCCCAT -3’
hnRNPA2/B1_ex9 F	5’- CAATTTTGGAGGTAGCCCTG -3’
hnRNPA2/B1_ex9 R	5’- CTCCATAGTTGTCATAACCACC -3’
ATP7A F	5’- ATTGATGACATGGGCTTTGA -3’
ATP7A R	5’- GCAATGTGCTTTGGATATGG -3’
hS18 F	5’- CTGCCATTAAGGGTGTGG -3’
hS18 R	5’- TCCATCCTTTACATCCTTCTG -3’
ATP7B F	5’-AGGAGCCCTGTGACATTCTT-3’
ATP7B R	5’-TTGCTCTTTGCCAAGTGTTC-3’
Ctr1 F	5’-GACCAAATGGAACCATCCTT-3’
Ctr1 R	5’-ATGACCACCTGGATGATGTG-3’
SOD1 F	5’-TGAAGAGAGGCATGTTGGAG-3’
SOD1 R	5’-ATGATGCAATGGTCTCCTGA-3’
**Plasmid construct primers**	
hnRNPA2/B1 R1	5’- AGCCATGGCAGCATCAAC -3’
hnRNPA2/B1 F1	5’- TAATGAGGGATCCTGCAAGC -3’
hnRNPA2/B1 F2	5’- AAAGAAGATACTGAGGAACATCACC -3’
hnRNPA2/B1 F4	5’- CTTTGGTGGTAGCAGGAACA -3’
hnRNPA2/B1 R4	5’- ACCCTGGTTGCCATATCCA -3’

Suppl. Table 3. Antibodies used in Western blotting and Immunofluorescence experiments.

Antibody	Catalog No.	Company	Dilution
hnRNPA2/B1	R4653-200UL	Sigma-Aldrich	1:1000 WB
CTPS2	ab196016	Abcam	1:10,000 WB
ATP7A	sc-376467	Santa Cruz	1:1000 WB, 1:200 IF
TGN46	GTX74290	Genetex	1:200 IF
Sheep anti-mouse IgG-HRP	AC111P	Sigma-Aldrich	1:5000 WB
Goat anti-rabbit IgG-HRP	sc-2004	Santa Cruz	1:5000 WB
AlexaFluor 488 donkey anti-rabbit	A-21206	Thermo Fisher	1:500 IF
AlexaFluor 568 donkey anti-mouse	A10037	Thermo Fisher	1:500 IF
AlexaFluor 568 donkey anti-sheep	A-21099	Thermo Fisher	1:500 IF

Supplementary Figure 1.

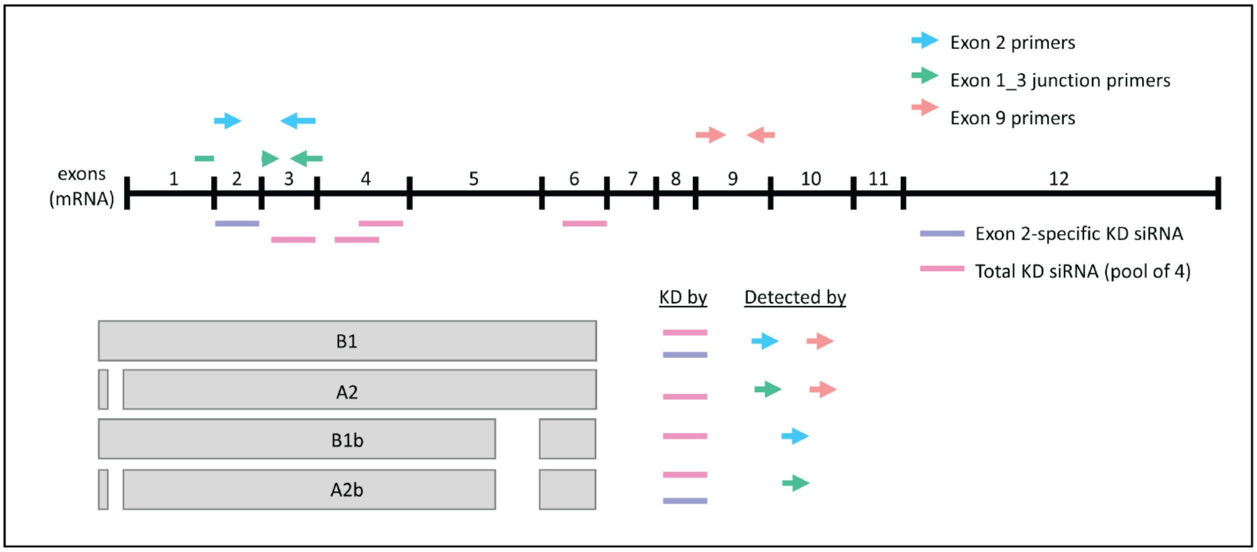

Supplementary Figure 2. Schematic of siRNA used to knockdown *hnRNPA2/B1* and primers used to detect *hnRNPA2/B1* isoform expression using qRT-PCR.

Schematic representation of the organization of the four hnRNPA2/B1 isoforms at the transcript-level. Exons 2 and 9 are alternatively spliced to generate the four isoforms: B1, A2, B1b, and A2b. Box length is indicative of the relative size of the exon within the transcript. Lines indicate where in the transcript the siRNA target. Target sites of the siRNA against all isoforms (total KD) are indicated in pink and the target site of the siRNA against exon 2 (ex2 KD) is indicated in purple. Arrows indicate the area in *hnRNPA2/B1* that is amplified by the primer. Primers that detect exon 2 (B1 and B1b) are in blue, primers that detect the exon 1_3 junction (A2 and A2b) are in green, and primers that detect exon 9 (B1 and A2) are in orange.

Supplementary Figure 2.

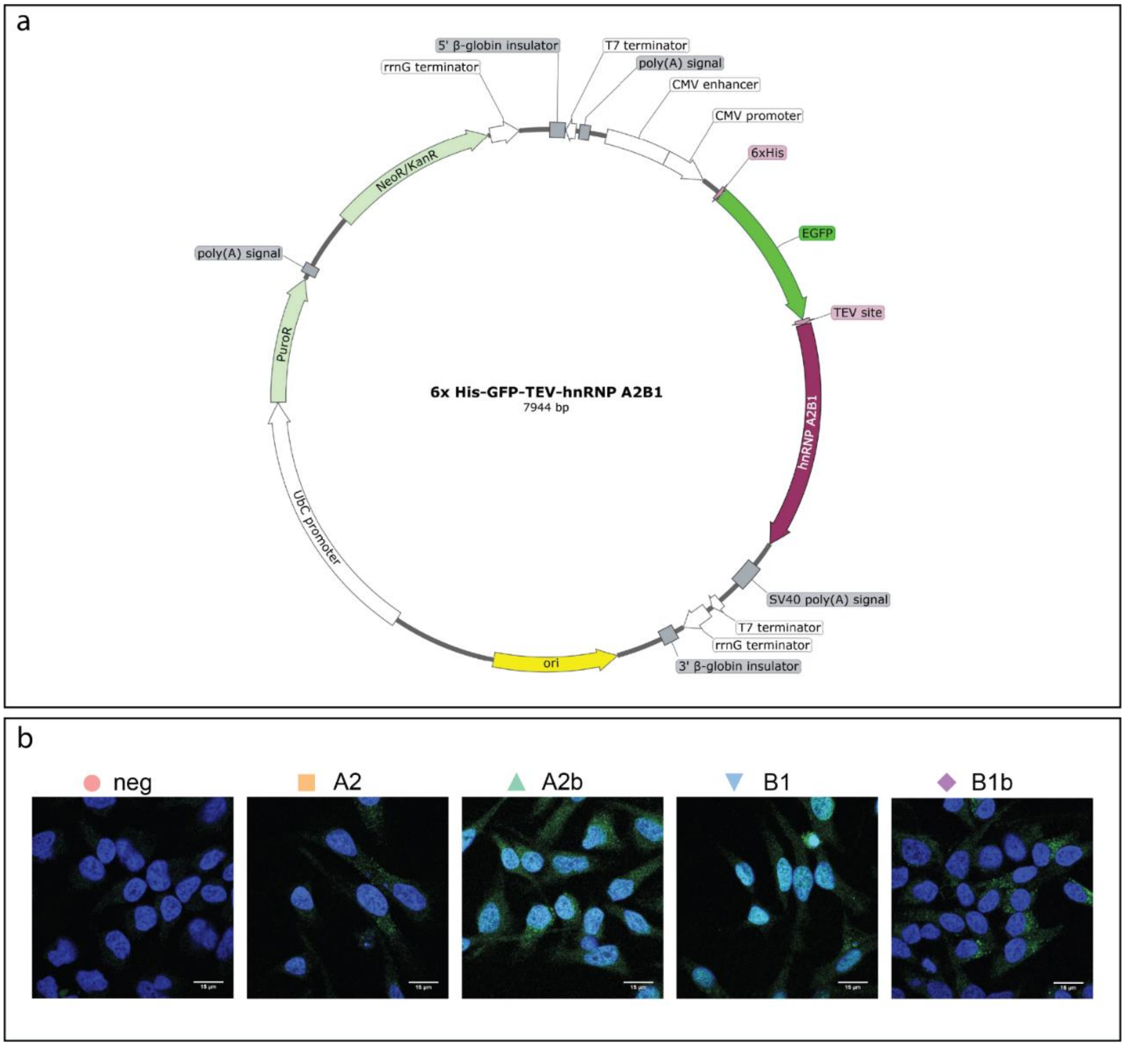

Supplementary Figure 3. hnRNPA2/B1 isoform plasmids used in overexpression experiments.

Each hnRNPA2/B1 isoform was cloned into a 6x His-GFP-TEV (HGT) plasmid backbone for expression in mammalian cells. **(a) Representative plasmid map of the HGT-hnRNPA2/B1 isoform plasmids.** Each isoform has an N-terminal 6x His and GFP tag linked via a TEV cleavage site. **(b) Expression of the HGT-hnRNPA2/B1 isoform plasmids in HeLa cells.** HeLa cells were transfected with either no plasmid (neg) or each of the individual HGT-hnRNPA2/B1 isoform plasmids (A2, A2b, B1, B1b) for 24 h. Intensity of the green GFP signal is indicative of expression level. The HGT-hnRNPA2/B1 plasmid signal is in green and the nucleus (DAPI) in blue.
